# Discrepancies between human and murine model cerebral aneurysms at single-cell resolution

**DOI:** 10.3389/fcell.2025.1512938

**Published:** 2025-03-11

**Authors:** Hang Ji, Guicheng Kuang, Hailan Yang, Haitao Liu, Yue Li, Shaoshan Hu, Anqi Xiao, Chao You, Haogeng Sun, Chaofeng Fan, Guozhang Sun

**Affiliations:** ^1^ Department of Neurosurgery, Sichuan University West China Hospital, Chengdu, China; ^2^ Department of Neurosurgery, Zhejiang Provincial People’s Hospital, Hangzhou, China; ^3^ Department of Neurosurgery, Hei Longjiang Provincial People’s Hospital, Harbin, Hei Longjiang, China

**Keywords:** cerebral aneurysm, human CA, murine model CA, single-cell RNA sequencing, inflammation

## Abstract

**Background:**

The murine model of cerebral aneurysm (CA) serves as a prevalent tool for investigating the molecular underpinnings of CA. However, the extent to which the CA murine model aligns with that of human remains elusive.

**Methods:**

The present study employed a comprehensive integration and exploration of the single-cell RNA-seq (scRNA-seq) datasets, along with multiple trajectory and gene regulatory network analyses, to investigate the cellular and molecular discrepancies between human and murine model CAs.

**Results:**

The uniform manifold approximation and projection (umap) embedding exhibits that the primary discrepancies between human and murine model CAs reside in the cells of modifiable phenotype, encompassing vascular smooth muscle cell (vSMC), monocyte/macrophage, and neutrophil. The vSMCs from human CA tissue exhibit a fibroblast-like phenotype in comparison to that of murine model. Distinct patterns of neutrophil recruitment are observed in human and murine models, with the former characterized by neutrophil-derived CXCL8 and the latter by monocyte/macrophage-derived CCLs. In addition, macrophages originated from human unruptured CA express higher levels of M2 gene markers. Moreover, the inflammatory status of the CA tissue differs between humans and mouse models, with the former exhibiting a more acute and intense inflammation.

**Conclusion:**

These findings demonstrate subtle but important disparities between human and murine model CAs, and may shed light upon an optimization of murine CA model.

## Introduction

Cerebral aneurysm (CA) is a common cerebral vascular disease affecting up to 5% healthy adults (Etminan and Rinkel, 2016). The aneurysmal subarachnoid hemorrhage (aSAH) caused by CA rupture is an extremely fetal condition, leading to 25% prehospital death and over 50% morbidity and mortality risk (Claassen and Park, 2022). Currently, the best practice for blocking aSAH is to manage the CA through either surgery clipping or endovascular occlusion before rupture, which is extremely effective and the risk of postoperative complications is minimal. Nevertheless, the substantial incidence and prevalence of CA along with the catastrophic consequences of aSAH prompt extensive investigations of the molecular underpinnings of CA formation, progression and rupture for the development of a more cost-effective management strategy.

The murine CA model serves as a critical medium for the investigation of the molecular underpinnings of CA. Currently, the prevailing CA model is based on murine saccular aneurysms. The primary methods of model construction involve the ligation of carotid artery and renal arteries, along with procedures for artificially inducing hypertension, resulting in the development of CA within several months (Morimoto et al., 2002). The local injection of elastase greatly expedited the process of CA formation on this basis (Hosaka et al., 2014). Notably, the murine CA model has been demonstrated in various studies to be a valuable tool for simulating the histological and cellular composition changes observed in human CA (Hosaka et al., 2014; Jin et al., 2022; Ruzevick et al., 2013). Nevertheless, the distinctive vascular structure and physiological environment of mice, along with the procedure aspects of surgically modeling human spontaneous CA, and even the variations in the duration of aneurysms *in vivo*, present potential challenges to the extent to which murine model CA can simulate human spontaneous CA.

In this perspective, we thoroughly explored the cellular and molecular discrepancies of saccular CA domes originated from human and murine model at single-cell resolution, and elucidates that the primary discrepancies are associated with cells of modifiable phenotypes and the overall inflammation status. This study may provide valuable insights for the improved development of murine saccular CA models.

## Materials and methods

### Sample collection and preprocessing

All experiments involving human patients were conducted in accordance with the ethical policies and procedures of the ethics committee of West China Hospital, Sichuan University (No. 2019-428, 2019-06-15) and in accordance with Good Clinical Practice guidelines and the World Medical Association Declaration of Helsinki. The collection, preprocessing, sequencing, and data management procedures of unruptured and ruptured CA domes have been described in detail previously (Ji et al., 2024a). The cell-to-gene expression matrix, feature and barcode files of unruptured and ruptured murine CA tissues were retrieved from the GEO data portal (GSE193533) (Martinez et al., 2022). Briefly, fifty-eight-week-old male C57/BL6 mice were pharmacologically induced hypertension through ligation of the left renal artery and the right internal carotid artery and stereotactically injected of elastase into the basal cistern 1 week after surgery. For the comparison of human and murine CA, we constructed standard unruptured and ruptured cerebral aneurysm atlases to avoid potential bias of cell counts.

### Construction of a standardized unruptured and ruptured CA atlas

We constructed a human Standardized Unruptured CA Atlas (SUCA) for the comparison with unruptured CA from murine model. The SUCA consists of a total of four unruptured CA of human adult origin (Supplementary Table S1). We conducted stringent quality control for each sample separately, with the criteria being set as 350 < nfeature <4,500, and the proportion of mitochondrial genes <35%. Doublets were excluded using the R package “DoubletFinder” (McGinnis et al., 2019). The samples are integrated by utilizing the “HarmonyIntegration” method of the “IntegrateLayers” function of the R package “Seurat” (v5.0) (Hao et al., 2023). We initially set the resolution to 0.35 for identifying cell communities. After assignment of cell types, we randomly selected a quarter of each type of cell for the composition of the SUCA. Similarly, a human Standardized Ruptured CA Atlas (SRCA) using two ruptured IA domes following the same criteria and approach (Supplementary Table S1).

### Integration of human and murine scRNA-seq datasets

The cell-by-gene expression matrix of sham (psudo-operation), formed (CA formation), and ruptured CA developed by Martinez AN et al. were retrieved from the Gene Expression Omnibus (GSE193533) (Martinez et al., 2022). The conversion of homologous genes of *mus musculus* and homosapiens were conducted using the R package ‘biomart’ (Durinck et al., 2009). Data integration was conducted using the ‘harmony’ algorithm implemented in the framework of ‘Seurat’ (v5.0) (Hao et al., 2023).

### Functional enrichment analysis

The differential expressed genes (DEGs) were calculated using the “FindAllMarkers” function of the “Seurat” R package with default parameter. The average log2 fold change >1, adjusted p value <0.05 and pct.1 > pct.2 was set as the cutoff. Functional enrichment analysis was conducted based on the Gene Ontology and Kyoto Encyclopedia of Genes and Genomes (KEGG) using the webtool Metascape (https://metascape.org/) (Zhou et al., 2019; Thomas et al., 2021; Goad and Kanehisa, 1982).

### Principal component analysis

The principal component analysis (PCA) was performed to explore the similarity of cells using the R function “prcomp.” The count matrix was log transformed and scaled. Top 8,000 variable genes were filtered through calculating the mean absolute deviation (MAD). The cell type average expression matrix was used as the input. The coefficient matrix and eigen vectors was calculated to avoid overweighting of an outlier.

### Trajectory analysis

Multiple trajectory analyses were conducted for dissecting the internal association between cells. Diffusion map, implemented in the R package “destiny,” leverages local similarity and nonlinear characteristics of data to preserve the intrinsic structure and continuity of the dataset, thereby effectively capturing the features inherent in its continuous distribution of cells (Angerer et al., 2016; Haghverdi et al., 2015). The monocle algorithm was additionally employed to further identify branches of the trajectory and generate pseudotime (Trapnell et al., 2014). Its main principle is to project high-dimensional gene expression data into a lower-dimensional space while preserving the complete structure of the data using the DDRTree algorithm.

### Cell-cell communication analysis

The interaction between cell types were inferred using the R package ‘CellChat’ (Luo et al., 2023). We extracted the count data and do normalization and log transformation as the input of CellChat. The CellChat database is a manually curated literature validated database of ligand receptor interactions in humans and mice, including autocrine/paracrine signaling interactions, extracellular matrix (ECM) receptor interactions, and cell-cell contact interactions. To ensure the reliability of the “computCommunProb” function, we set the truncation value of ‘trimean’ to 0.25, meaning that if the percentage of a group of expressing cells is less than 25%, the average gene expression is defined as 0. The p value of cell-cell communication was adjusted using the false discovery rate (FDR).

### Gene regulatory network analysis

The transcriptional gene regulatory network (GRN), the transcription factors (TFs) and their target genes, was inferred using the R package “SCENIC” (Bravo González-Blas et al., 2023). Genome reference files of human (hg38) and mice (mm10) were retrieved from the CisTarget database (https://resources.aertslab.org/cistarget/). The filtering criterion is set as the sum of gene expression levels >3% of the total number of cells, and it should be expressed in over 1% of the cell population. The co-expression modules were constructed by grouping genes with weight >0.001 (w001) around each TF.

### Immunofluorescence staining

Multiplex immunofluorescence staining (mIHC) was performed on sections of a giant unruptured IA sample. Fresh IA domes were fixed in 4% paraformaldehyde and dehydrated in graded methanol overnight at −20°C. Samples were rehydrated, washed, and blocked with SEA BLOCK Blocking Buffer sequentially, followed by incubation with primary antibodies overnight at 4°C. Anti-CD31 (1:1000; HUABIO, China), anti-αSMA (1:2000; HUABIO, China), anti-CD68 (1:1000; HUABIO, China), anti-CD11b (1:1000; HUABIO, China), and anti-DCN (1:500; HUABIO, China) were employed to determine the expression of proteins of interest. The second antibodies used were rabbit poly-HRPs labeled with cyclic-480/550/630 dyes using IRISKit HyperView mIF kit (LUMINIRIS, China). DAPI was used as the nuclear counterstain. The slices were scanned using the Olympus VS200 scanner. The images were adjusted using ImageJ followed by analyzing using the QuPath software version 0.4.4.

### Statistics

All statistics was performed using the R software (v4.3.3). Mann-Whiney U rank sum test was conducted for the calculation of DEGs, and p values were adjusted through applying false discovery rate. An adjusted p value less than 0.05 was defined as statistically significant.

## Results

### Overview of unruptured and ruptured CA from human and murine model

**FIGURE 1:**
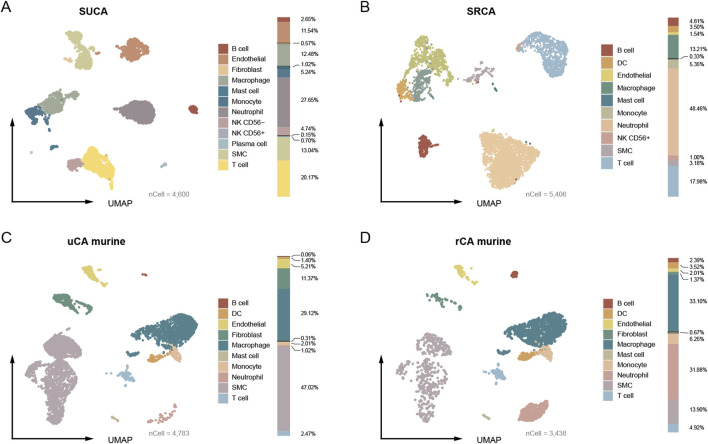
The umap embeddings of CA from human and murine model. **(A)** The umap embedding of human standard unruptured CA (SUCA). **(B)** The umap embedding of human standard ruptured CA (SRCA). **(C, D)** The umap embedding of unruptured and ruptured CA originated from murine model.

The SUCA is constructed using four unruptured CA domes and comprises of a total of 4,600 quantified cells, including endothelial (n = 531, 11.54%), vSMC (n = 600, 13.04%), fibroblast (n = 26, 0.57%), lymphocytes (T cell, B cell, plasma cell, CD56^+^ NK cell and CD56^−^ NK cell, n = 1,307, 28.41%), neutrophil (n = 1,272, 27.65%), monocyte (n = 241, 5.24%), macrophage (n = 574, 12.48%), and mast cell (n = 49, 1.07%). The proportion of each type of cell is exhibited (Figure 1A). No dendritic cell (DC) is found in the unruptured human CA. In turn, the SRCA comprises of 5,406 quantified cells, including endothelial (n = 83, 1.54%), vSMC (n = 172, 3.18%), lymphocytes (T cell, B cell, and NK cell, n = 1,321, 24.44%), neutrophil (n = 2,620, 48.46%), monocyte (n = 289, 5.35%), macrophage (n = 714, 13.21%), DC (n = 189, 3.50%), and mast cell (n = 18, 0.33%) (Figure 1B). No endothelial cell and fibroblast are captured in the ruptured human CA, which is consistent with the severe damage to the artery wall caused by an aneurysm rupture (Etminan and Rinkel, 2016). Overall, the proportion of vascular cells, including endothelial, vSMC, and fibroblast, decreased markedly in rupture CA (unruptured/ruptured: 25.15% vs. 4.72%), while the proportion of neutrophil is remarkably increased (unruptured/ruptured: 27.65% vs. 48.46%).

As for CA tissue from murine model, a total of 4,783 quantified cells are captured from the unruptured CA, encompassing endothelial (n = 249, 5.21%), vSMC (n = 2,249, 47.02%), fibroblast (n = 544, 11.37%), lymphocytes (B cell and T cell, n = 121, 2.53%), neutrophil (n = 49, 1.02%), monocyte (n = 96, 2.01%), macrophage (n = 1,393, 29.12%), DC (n = 67, 1.40%) and mast cell (n = 15, 0.31%) (Figure 1C). The ruptured murine CA contains 3,438 quantified cells, including endothelial (n = 69, 2.01%), vSMC (n = 478, 13.90%), fibroblast (n = 47, 1.37%), lymphocytes (B cell, T cell, n = 251, 7.30%), neutrophil (n = 1,096, 31.88%), monocyte (n = 215, 6.25%), macrophage (n = 1,138, 33.10%), DC (n = 121, 3.52%), and mast cell (n = 23, 0.67%) (Figure 1D). A remarkable decreasing of the vascular cells (endothelial, vSMC, and fibroblast) in the ruptured murine CA is also observed (63.6% vs. 17.28%). Notably, the proportion of vascular cells of unruptured CA of human patients were lower than that of murine model (25.15% vs. 63.6%), which may be ascribed to over 2,000 vSMCs (47.02%) are captured in the unruptured CA from murine model. The most significant decrease is observed in the proportion of vSMC (human, 9.86% reduction; murine model, 33.12% reduction). Additionally, the unruptured CA of human patients contained remarkably higher proportion of neutrophil than that of murine model (27.65% vs. 1.02%), which plays a crucial role in facilitating CA rupture (Korai et al., 2021). Therefore, these results demonstrate preliminary discrepancies between human and murine model CAs.

### Construction of an integrated cell atlas

**FIGURE 2:**
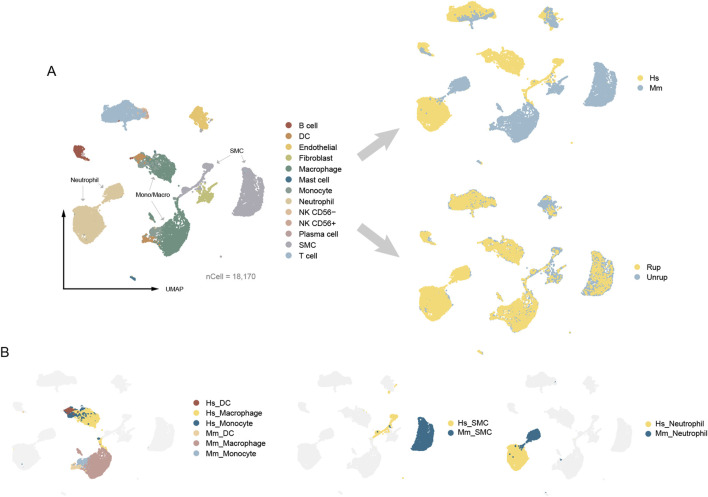
The umap embeddings of integrated CA atlas. **(A)** The integrated CA atlas. **(B)** The distribution of cell types with modifiable phenotypes. Hs: *homo sapiens*, Mm: *mus musculus*.

The construction of an integrated CA atlas of human and murine model is carried out according to stringent criteria. We performed independent quality control for each dataset. To maximize the retention of vSMCs, we limited the mitochondrial proportion to ≤35%. To minimize the presence of deceased cells or cell debris, we employed different resolutions (0.5, 1.0, and 1.5) for cell community detection, resulting in a final identification of 9, 14, and 20 clusters. We excluded cell clusters exhibiting high expression of ribosomal transcripts, such as *RPL32*, *RPL13*, and *RPL18A*, which accounted for 0.31% of all cells. As a result, a total of 18,170 cells were assigned, including endothelial (n = 849), vSMC (n = 3,499), fibroblast (n = 617), lymphocyte (T cell, B cell/plasma cell, NK cell, n = 2,965), mono/macro (monocyte, macrophage, DC, n = 5,027), neutrophil (n = 5,107), and mast cell (n = 106) (Figure 2A). There are cell types in humans and murine model that can be projected into a common community in the umap embedding, including endothelial cells, lymphocytes (T cell, B cell and NK cell), and mast cells, indicating conserved gene expression patterns. Intriguingly, there are also cell types classified into distinct communities with a proximal distance in the umap embedding, encompassing vSMC, neutrophil, and mono/macro (monocyte, macrophage, DC) that possess modifiable phenotypes (Alencar et al., 2020; Salcher et al., 2022; Xue et al., 2014) (Figure 2B). Collectively, these findings suggest that the potential discrepancies between human and murine models of CA may be attributed, at least in part, to cells of modifiable phenotype.

### The transcriptomic conservation of endothelial cells

**FIGURE 3:**
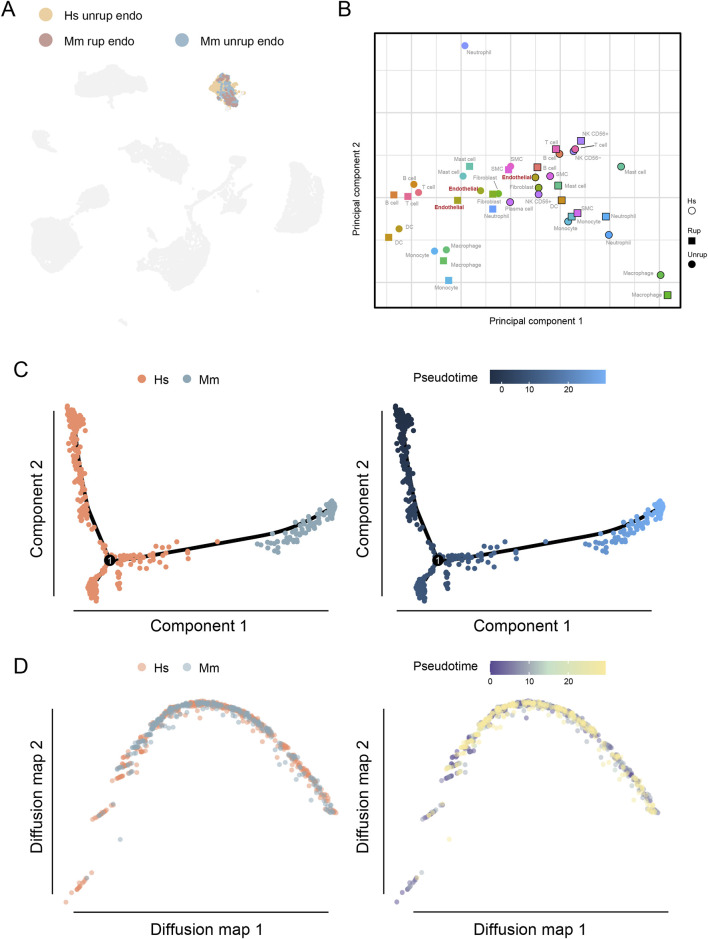
Endothelial cells in the human and murine CA model. **(A)** The umap embedding of human and murine model endothelial cells. **(B)** PCA analysis of cell types of different origin. **(C)** DEGs of endothelial cells from unruptured CA of human and murine model. **(D)** Functional enrichment analysis. Hs, *homo sapiens*; Mm, *mus musculus*.

The endothelial cell dysfunction plays a critical role in the formation and progression of CA (Etminan and Rinkel, 2016; Turjman et al., 2014; Frösen et al., 2012), we therefore characterized the transcriptomic features of human and murine model endothelial cells. As a result, a total of 531 endothelial cells were identified in human unruptured CA and 318 from murine model, accounting for 5.34% and 3.87% of the total cell population, respectively. These cells can be projected into a common umap community (Figure 3A). Consistent with these findings, the PCA analysis shows the spatial proximity of endothelial cells, further indicating conserved transcriptomic features (Figure 3B). In addition, multiple trajectory analyses were performed for further dissecting the potential disparities, including the monocle that is sensitive to branches of trajectory and the diffusion map that better reflecting the continuity of single cells. Despite the observed disparities between human and murine model CA endothelial cells along the pseudotime in monocle, the findings of diffusion map do not provide support for such discrepancies (Figures 3C, D). Therefore, these findings may suggest that the transcriptomes of endothelial cells in human and murine model of CA are conserved.

### The transcriptome discrepancies in vSMCs

**FIGURE 4:**
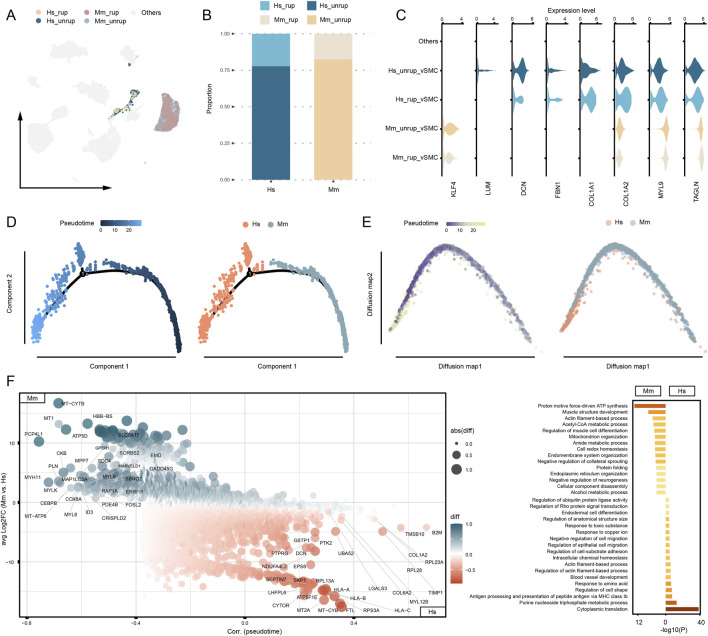
Phenotype discrepancies between human and murine model vSMCs. **(A)** The proportion of vSMCs captured from human and murine model. **(B)** Expression of SMC and fibroblast marker genes. Others refers to cells that are distinct from vSMCs. **(C)** Disparities of gene expression between human and murine vSMCs. **(D)** Trajectory analysis based on monocle. **(E)** Trajectory analysis based on diffusion map. **(F)** DEGs and functional enrichment analysis. Hs: *homo sapiens*, Mm: *mus musculus*.

Recent studies highlight the modifiable phenotype of vSMCs that are involved in various cardiovascular and cerebral vascular diseases (Yap et al., 2021; Sorokin et al., 2020; Lei et al., 2023). In our study, a total of 772 vSMCs from human CA samples and 2,727 from murine model are designated as distinct communities in the umap embedding, with the majority of the cells originating from unruptured CA (81.42%) (Figures 4A, B). The umap embedding shows a closer proximity between human vSMCs and fibroblasts (Figure 2A), which is corroborated by the observation that human vSMC express higher levels of fibroblast marker genes, including *COL1A1/2*, *FBN1*, and *DCN* (Figure 4C; Supplementary Figure S1). To further understand the disparities between human and murine model vSMCs, the trajectory analyses were performed. As a result, both monocle and diffusion map analyses reveal a significant uneven distribution of human and murine model vSMCs along the trajectory, with a notable enrichment of human vSMCs toward the terminal stage of pseudotime (Figures 4D, E). To unbiasedly understand the gene expression features associated with vSMC heterogeneity, we combined the calculation of DEGs and their correlation with our constructed pseudotime. As a result, the well-defined vSMC gene marker gene *MYH11* is highly expressed by murine model vSMCs, as well as other myosin encoding genes such as *MYL6* and *MYL9* (Figure 4F). Instead, the vSMC cells from human CA highly express genes associated with extracellular matrix (*COL6A2*, *COL1A2*, *LGALS3*), cytoskeleton remodeling (*TMSB10*, *DCN*), and HLA class I molecule coding genes that are involved antigen presentation (*HLA-A*, *HLA-B*, *HLA-C*). Moreover, the purine nucleoside triphosphate metabolic process is enriched in human vSMCs (Figure 4F), which is associated with cell proliferation upon pathological stimulus (Ma et al., 2023; Ma et al., 2022). Given the alternative phenotypes of vSMCs, these results demonstrate a deviation of human vSMC phenotype from normal contractile vSMCs.

### The different pattern in neutrophil recruitment and activation

**FIGURE 5:**
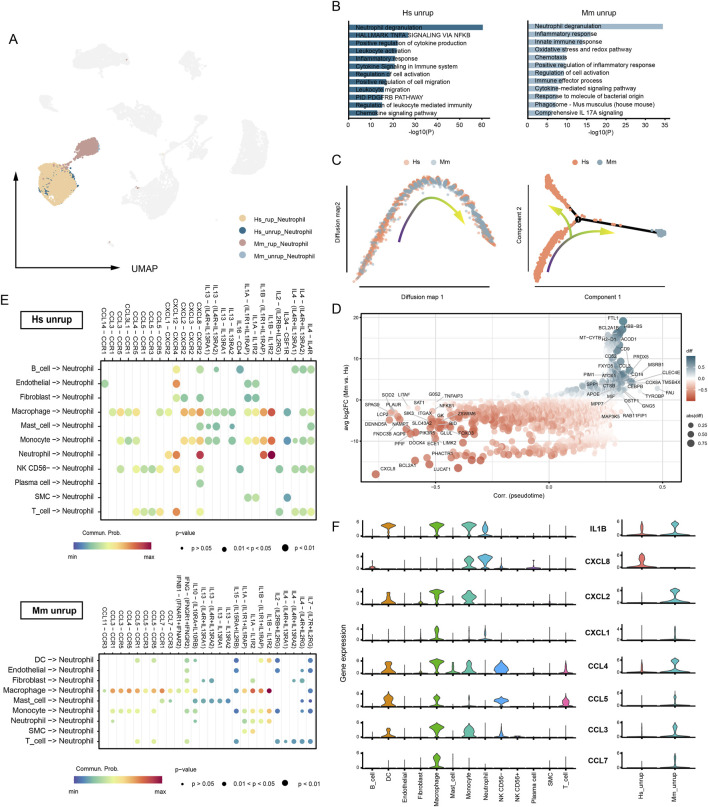
Recruitment of neutrophil in human and murine model CA. **(A)** The umap embedding of neutrophils from human and murine model. **(B)** Functional enrichment analysis. Neutrophil of human unruptured CA origin vs. neutrophil of murine model unruptured CA origin. **(C)** Trajectory analysis based on diffusion map (left) and monocle (right). **(D)** DEGs between human and murine. **(E)** Cell-cell communication analysis. Source cell, all types of cells. Target cell, neutrophil. Interaction type, cell-cell contact and secreted signaling. **(F)** The expression of neutrophil recruitment cytokines and chemokines. Hs, *homo sapiens*; Mm, *mus musculus*.

Neutrophil infiltration is destructive to the arterial wall and is closely associated with CA rupture (Ji et al., 2024a; Korai et al., 2021; Ji et al., 2024b). The presence of neutrophils in unruptured CA is observed in both human (27.65% of total cell) and murine model (1.02%), with a significant increase upon CA rupture (48.68% in humans and 31.88% in murine model) (Supplementary Figure S1). Neutrophils of human and murine model origin are presented as two related communities in the umap embedding (Figure 5A). The functional enrichment analysis shows that the most enriched term in neutrophils is degranulation in both groups (Figure 5B). Intriguingly, the pseudotime analyses suggest discrepancies in human and murine model neutrophils. The diffusion map suggests a biased distribution of cells along the trajectory, with human neutrophils enriched in one end of the trajectory (Figure 5C). Subsequently, the monocle algorithm detected branches of the trajectory, which further characterizes the disparities (Figure 5C). Genes that are highly associated with the pseudotime and differentially expressed in human and murine model are calculated (Figure 5D). For instance, *FTL1*, involved in iron storage; *ACOD1*, involved in metabolic regulation; and *TMSB4X* and *CCL3*, involved in neutrophil migration and chemotaxis, are significantly enriched in murine model neutrophils. Instead, human CA neutrophils highly express *CXCL8* and *DOCK4* that regulating chemotaxis and migration. These findings may also suggest distinct patterns of neutrophil infiltration in human and murine model, prompting us to conduct a cell-cell communication analysis to gain further insights into the ligand-receptor-based interactions involved in neutrophil recruitment. Generally, monocyte/macrophage and neutrophil exhibit potent interactions with neutrophils, especially through chemokines and IL1β (Figure 5E). In human CA, the recruitment and chemotactic action of neutrophils towards themselves is remarkably CXCL8-dependent, while in the murine model, neutrophil recruitment is primarily mediated by CCLs (Figure 5F). Thus, the pattern of neutrophil recruitment in human and murine model may be different.

### The discrepancies in macrophage activation program

**FIGURE 6:**
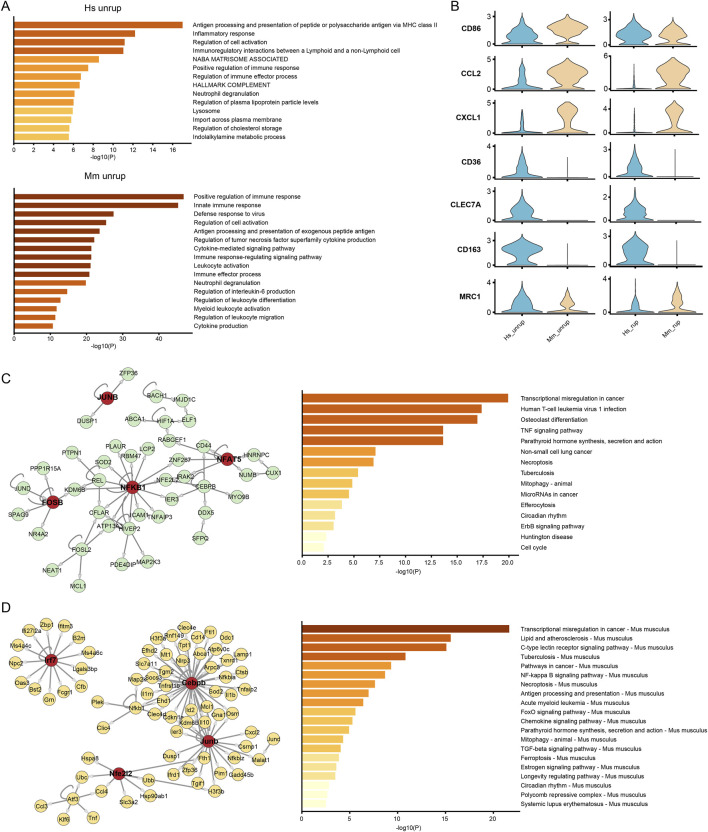
Activation program of macrophages. **(A)** Functional enrichment analysis of macrophages from unruptured human and murine model CA. **(B)** The expression of M1 (CD86, CCL2, CXCL1) and M2 (CD36, CLEC7A, CD163, MRC1) type marker genes. **(C, D)** The GRN and enrichment analysis of high-confidential target genes of macrophage from unruptured human and murine model CA, respectively. Hs: *homo sapiens*, Mm: *mus musculus*.

Macrophages play a paramount role in CA inflammation (Etminan and Rinkel, 2016). The proportion of macrophages in the SUCA was 12.48%, which increased to 13.21% in the SRCA (Supplementary Figure S1). In the murine model, there are higher proportion of macrophages, with proportions of 29.12% and 33.10% being observed in the unruptured and ruptured CA tissues, respectively. The functional enrichment analysis of DEGs indicates that terms associated with antigen presentation, immune response, and regulation of immune cells are significantly enriched in macrophages of human unruptured CA (Figure 6A), which are similar to that of macrophages from unruptured murine model. Nevertheless, macrophages from human unruptured CA express higher levels of M2 marker genes (*CD36*, *CLEC7A*, *CD163* and *MRC1*) and lower levels of M1 marker genes (*CD86*, *CCL2*, *CXCL1*) than that of murine mode (Figure 6B), indicating a preference of M2 polarization of human CA macrophages. Further, we investigated the gene regulatory network underline these disparities. As a result, macrophages from unruptured human CA are characterized by the transcriptional activation of NF-κB, FOS and JUN, and their high-confidential target genes are enriched in pathways including TNF signaling pathway (Figure 6C). In turn, the macrophages from murine model are governed by TFs including Cebpb, Junb, and Nfe2l2. Particularly, the irf7 is associated with M1 polarization of macrophage (Ling et al., 2023). Moreover, functional enrichment analysis suggests pathways enriched in murine CA macrophages, including lipid and atherosclerosis and FoxO (Figure 6D), where the FoxO pathway is involved in the M1 towards M2 phenotype polarization (Chung et al., 2015).

### Differences of the overall inflammation status

**FIGURE 7:**
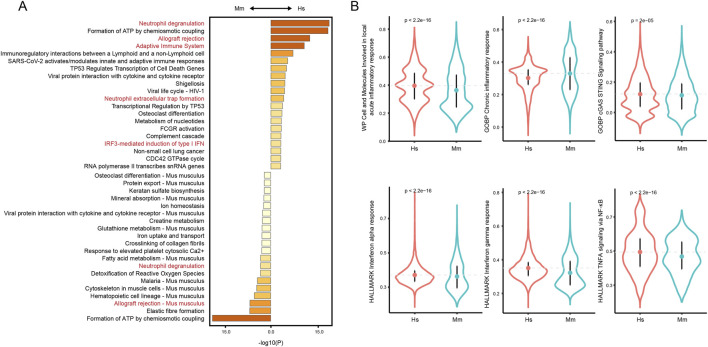
Overall inflammation status of human and murine model CA. **(A)** Functional enrichment analysis of filtered DEGs. **(B)** Comparison of ssGSEA scores of inflammation-related pathways.

Given the crucial role of inflammation in CA rupture and our finding that immune cells such as neutrophils and monocyte/macrophages in human and murine model CA remain distinct, we next characterized the overall disparities of the local inflammation status. To rigorously filter genes for downstream analysis, we eliminated transcripts expressed in fewer than 10 cells. Next, we selected genes highly variable between cells through retain those transcripts with a residual variance over 0.5, filtered down to 6,374 genes for further analysis. The DEGs were calculated and functional enrichment analysis suggests differences in immune-associated pathways. Despite that neutrophil degranulation and allograft rejection are enriched in both groups, terms including adaptive immune system, neutrophil extracellular trap formation, and IRF3-mediated induction of IFN I are enriched in human CA tissue (Figure 7A), indicating disparities in immune status. Further, we calculated the ssgsea score of several inflammation-related pathways, including acute and chronic inflammation response, cGAS sting pathway, interferon alpha and gamma, and TNF alpha signaling pathway. The cGAS-STING pathway is triggered by the release of free DNAs from damaged cells (Ablasser and Chen, 2019), a phenomenon commonly observed in CA tissue. The TNF alpha, interferon alpha and gamma are important pro-inflammatory cytokines that are involved in the activation of multiple immune cells (Zhang et al., 2016; Sathyan et al., 2015). As a result, the human CAs score significantly higher in cells and molecules involved in local acute inflammatory response (Figure 7B), indicating a feature of acute inflammation. Notably, the ssgsea scores of cGAS-STING pathway, interferon alpha and gamma responses, and TNF alpha signaling *via* NF-κB are all higher in the human CA tissue, indicating a more intense inflammation in human CA tissue.

## Discussion

The CA remains a prevalent cerebral vascular disease that can pose a significant threat to human health. In this study, we integrated scRNA-seq data from human and murine CA domes and found differences in the gene expression programs exclusively in cell types of modifiable phenotype, including vSMC, monocyte/macrophages, and neutrophils. Besides, the overall inflammation status of human and murine model CA remains different. These findings may shed light upon the molecular discrepancies between murine model and human CA, and provide additional insights into the improvement of current murine CA model.

The natural history of human saccular CA and the molecular mechanisms underlying their occurrence and progression are intricate and not yet fully elucidated. The formation of CA may be attributed to endothelial cell dysfunction mediated by hemodynamic disturbance (Etminan and Rinkel, 2016; Turjman et al., 2014). The early pathological changes in human CA remain elusive due to the challenges in sample acquisition. The animal-based temporal model proposes that inflammation is linked to the arterial extracellular matrix remodeling (Frösen et al., 2012; Sluijter et al., 2004). In addition, studies based on magnetic resonance imaging suggest the macrophage infiltration and presence of local inflammation at the early stage (Shimizu et al., 2019). The local inflammation may be a consequential manifestation of hemodynamic disturbance and plays a pivotal role in the progression of CA (Etminan and Rinkel, 2016). Recently, our studies based on scRNA-seq analysis revealed a diverse repertoire of immune cells infiltrating the local unruptured CA wall, encompassing T/B lymphocytes, NK cells, granulocytes, mast cells alongside macrophages (Ji et al., 2024a; Ji et al., 2024b). The infiltration of these immune cells is associated with the degradation and remodeling of the ECM, as well as loss of endothelial cells and vSMCs. This process increases the vulnerability of the artery wall, thereby promoting the progression and rupture of CA. Nevertheless, further investigation is needed to determine whether the development of large, thick-walled CA is linked to persistent chronic inflammation, particularly its reparative branches.

The murine CA model is cost-effective and replicable, and the technique of construction has undergone several iterations. A previous method harvest aneurysm through the injection of elastase extravascularly to the carotid artery in mice (Hoh et al., 2010). Around the same time, researchers were able to induce CA in mice through artificially induced hemodynamic disturbances (Morimoto et al., 2002). The subsequent incorporation of elastase and hemodynamic disturbance further enhanced the robustness of the CA model and significantly reduced the modeling cycle (Hosaka et al., 2014). Despite the shortened modeling cycle may affect local inflammatory features, histological-based studies have demonstrated the presence of abundant macrophages and CD45^+^ leukocytes in the dome of modified CA murine model, exhibiting a resemblance to human CA (Frösen et al., 2012). Further, the scRNA-seq-based study demonstrated a remarkable diversity of immune cell types in the murine model CA (Martinez et al., 2022). Consistently, our integrated analysis revealed that the murine model CA exhibits a comparable major population of cells to that of the human, thereby further validating the robustness of the modeling approach.

The disease process of murine model CA, however, significantly diverges from that observed in human CA. For instance, the vascular structure and physiological environment in mice are significantly different from those in humans. The vascular walls in mice are thinner, and blood pressure levels are different, which may affect the formation and development of aneurysms. In addition, while murine models are typically induced to develop aneurysms through surgical procedures and drug administration, the pathogenesis of human aneurysms is predominantly influenced by a combination of genetic, environmental, and lifestyle factors. This inherent complexity and diversity in human aneurysm formation may limit the ability of murine models to fully recapitulate the intricacies. So, to what extent do they exhibit similarities? We found that the differences between murine model and human CA were mainly reflected in the cells with phenotypic plasticity, encompassing vSMC, monocyte/macrophage, and neutrophil. Human CA vSMC showed fibroblast-like phenotype, and macrophages expressed higher M2 marker genes. These alterations may be a sign of inflammatory tissue remodeling, which can be triggered by the recognition of damage-associated molecular patterns (Medzhitov, 2021). For instance, mitochondrial DNA serves as a potential biomarker of aneurysmal subarachnoid hemorrhage (Chaudhry et al., 2019). The high prevalence of CA and the annual rupture risk of approximately 1% result in patients experiencing a long lifespan carrying silent CA. In turn, the construction of a murine CA model takes 2–4 weeks, resulting in a distinct inflammatory environment. We assume that this is one of the crucial factors affecting the discrepancies between the cell status of murine model and the human CA.

Another interesting finding of this study is the different patterns of neutrophil recruitment. The infiltration of neutrophils in the CA tissue can be triggered by local activated platelets, monocyte/macrophages, and neutrophils themself (Capucetti et al., 2020). We observed that neutrophil recruitment in human CA tissue is CXCL8-dependent, whereas it relies on CCLs in the murine model. In mice, the direct equivalent of CXCL8 is absent, instead being substituted by CXCL1, CXCL2, and CXCL5 (Capucetti et al., 2020). These murine chemokines exhibit distinct structural and functional characteristics compared to their human counterpart, CXCL8. The CXCL8 plays a pivotal role in inflammatory responses by not only chemoattracting neutrophils but also activating and augmenting their bactericidal capacity, whereas CXCL1 and CXCL2 primarily participate in the early phase of inflammation by promoting neutrophil migration and aggregation (Han et al., 2021). Collectivey, it is noteworthy an augmented prominence of neutrophil-mediated inflammatory responses in human CA tissues, as we have observed a higher prevalence of neutrophils in human CA. Consequently, investigations relying on murine CA models are prone to underestimate the detrimental impact of neutrophils on the arterial wall.

There are some limitations in our study. Due to the challenges in constructing a CA murine model and the sparse cell population, application of single-cell sequencing is actually challenging. In addition to our in-house human CA scRNA-seq datasets, there is currently a lack of a large-scale murine model-originated data, which hampers the comparability between groups.

Taken together, we compared murine model and human CA at high resolution. Our findings reveal subtle discrepancies between human and murine CA, and may further shed light upon the optimization of the murine CA model.

## Data Availability

The raw data supporting the conclusions of this article will be made available by the authors, without undue reservation.

## Abbreviations

aSAH, aneurysmal subarachnoid hemorrhage; CA, cerebral aneurysm; umap, uniform manifold approximation and projection; DEG, differential expressed gene; ECM, extracellular matrix; GRN, gene regulatory network; KEGG, Kyoto Encyclopedia of Genes and Genomes; MAD, mean absolute deviation; PCA, principal component analysis; SUCA, standardized unruptured CA atlas; SRCA, standardized ruptured CA atlas; TF, transcription factor; vSMC, vascular smooth muscle cell.
