# Supplementary material for: Discrepancies between human and murine model cerebral aneurysms at single-cell resolution
Source: Front Cell Dev Biol. 2025 Mar 11;13:1512938. doi: 10.3389/fcell.2025.1512938 (PMC11933115; doi:10.3389/fcell.2025.1512938)
Supplement: Supplementary file 1 [file Table1.docx]

**Table S1. Sample information of CA tissues of human patient origin**

| **Group** | **Age** | **Gender** | **Diagnosis** | **Shape** | **Location** |
| --- | --- | --- | --- | --- | --- |
| uIA | 58 | Male | CA | Saccular | BA |
| uIA | 63 | Female | CA | Saccular | MCA |
| uIA | 58 | Male | CA | Saccular | MCA |
| uIA | 52 | Female | CA | Saccular | MCA |
| rIA | 41 | Female | aSAH | Saccular | PICA |
| rIA | 47 | Male | aSAH | Saccular | ACoA |
