# Supplementary material for: Discrepancies between human and murine model cerebral aneurysms at single-cell resolution
Source: Front Cell Dev Biol. 2025 Mar 11;13:1512938. doi: 10.3389/fcell.2025.1512938 (PMC11933115; doi:10.3389/fcell.2025.1512938)
Supplement: Supplementary file 2 [file DataSheet1.docx]

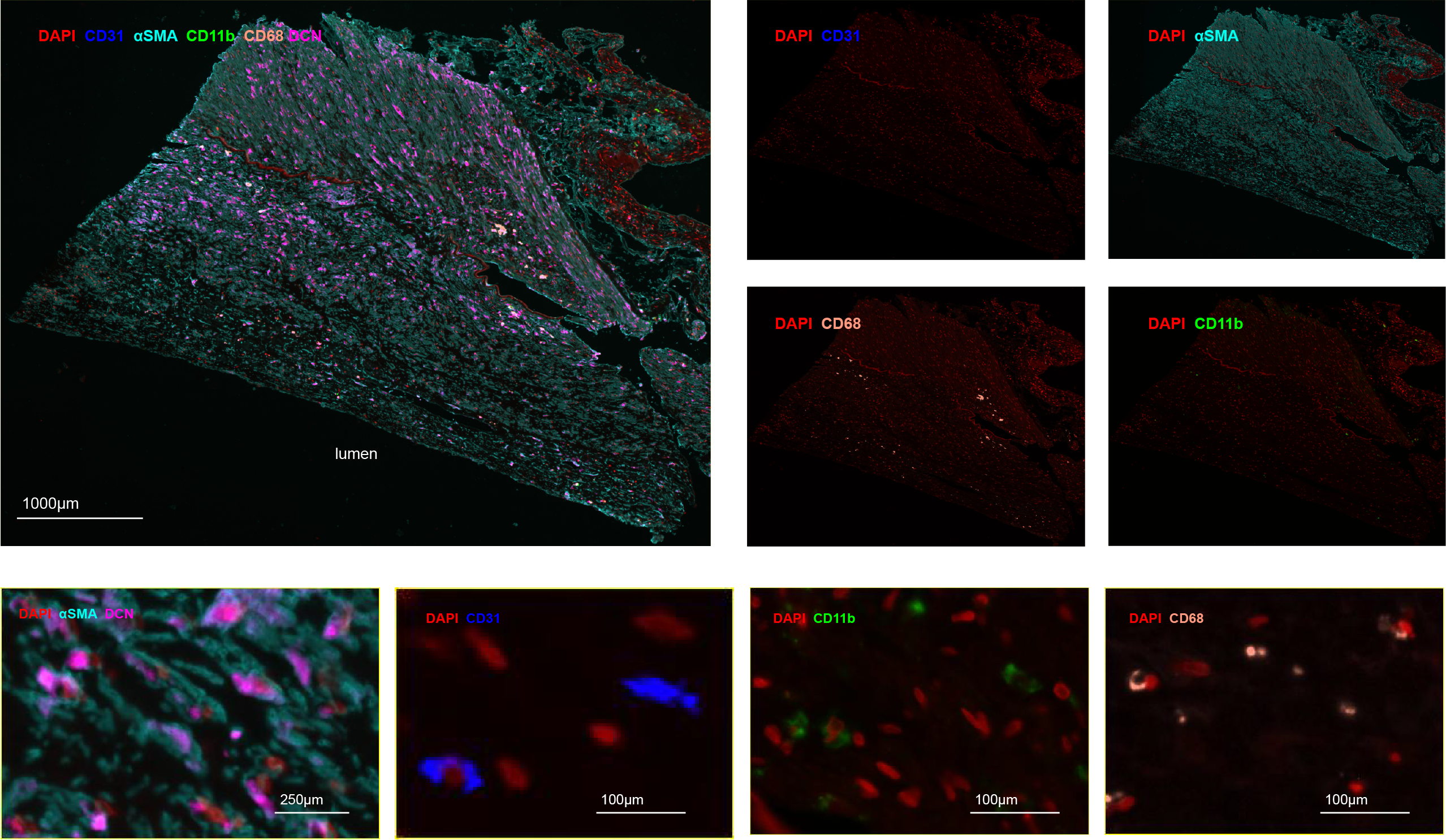


**Figure S1. mIHC validation of cells detected in human IA.** This sample represents the dome of a giant, unruptured saccular IA that lacks endothelial cells on the lumen surface. CD31 staining can be found in the IA wall. CD68 and CD11b positive cells were also detected, suggesting the infiltration of mono/macros and neutrophils. Some αSMA positive cells were also DCN positive, indicating the expression of fibroblast markers by vSMC.
